# High-Throughput, Label-Free Isolation of White Blood Cells from Whole Blood Using Parallel Spiral Microchannels with U-Shaped Cross-Section

**DOI:** 10.3390/bios11110406

**Published:** 2021-10-20

**Authors:** Amirhossein Mehran, Peyman Rostami, Mohammad Said Saidi, Bahar Firoozabadi, Navid Kashaninejad

**Affiliations:** 1 School of Mechanical Engineering, Sharif University of Technology, Tehran 11155, Iran; amirhossein.mehran@alum.sharif.edu (A.M.); peyman.rmi@gmail.com (P.R.); mssaidi@sharif.edu (M.S.S.); 2 Queensland Micro- and Nanotechnology Centre, Nathan Campus, Griffith University, 170 Kessels Road, Brisbane, QLD 4111, Australia

**Keywords:** WBC isolation, spiral microchannels, inertial microfluidics, passive cell separation, high-throughput separation

## Abstract

Rapid isolation of white blood cells (WBCs) from whole blood is an essential part of any WBC examination platform. However, most conventional cell separation techniques are labor-intensive and low throughput, require large volumes of samples, need extensive cell manipulation, and have low purity. To address these challenges, we report the design and fabrication of a passive, label-free microfluidic device with a unique U-shaped cross-section to separate WBCs from whole blood using hydrodynamic forces that exist in a microchannel with curvilinear geometry. It is shown that the spiral microchannel with a U-shaped cross-section concentrates larger blood cells (e.g., WBCs) in the inner cross-section of the microchannel by moving smaller blood cells (e.g., RBCs and platelets) to the outer microchannel section and preventing them from returning to the inner microchannel section. Therefore, it overcomes the major limitation of a rectangular cross-section where secondary Dean vortices constantly enforce particles throughout the entire cross-section and decrease its isolation efficiency. Under optimal settings, we managed to isolate more than 95% of WBCs from whole blood under high-throughput (6 mL/min), high-purity (88%), and high-capacity (360 mL of sample in 1 h) conditions. High efficiency, fast processing time, and non-invasive WBC isolation from large blood samples without centrifugation, RBC lysis, cell biomarkers, and chemical pre-treatments make this method an ideal choice for downstream cell study platforms.

## 1. Introduction

In medical science, the study of characteristics and mechanisms of cell functions, identifying external damaging factors, and finding methods to prevent and treat cellular disorder diseases in the human body requires the preparation of cell samples with suitable purity. Blood is the most important component of the human body, containing various types of essential cells, including red blood cells (RBCs), white blood cells (WBCs), and platelets which are produced in the bone marrow and released into the bloodstream [1]. WBCs comprise about 1% of whole blood and have a significant role in the body’s immune system. The change in the total number of WBCs in the blood is the harbinger of infections, autoimmune reactions, and other malignancies [2]. For example, in blood cancer diseases, such as leukemia and myeloma, the life cycle of normal blood cells is interrupted by the abnormal growth in the number of WBCs in the bone marrow and bloodstream. Hence, enumeration and monitoring of WBCs are critical in diagnosing various kinds of diseases [3]. Since RBCs comprise the majority of whole blood, complete or fractional separation of RBCs from a blood sample is usually required for subsequent cellular and molecular examination of WBCs. However, conventional cell sorting techniques, such as density gradient centrifugation, RBC lysis, porous filtration, and other methods are often labor-intensive, require a large volume of sample, and sometimes use expensive particular biomarkers or labels to identify the target cells [4].

In recent years, various microfluidic cell separation devices have been developed to minimize the amount of required sample, processing time, and operator-based errors while maintaining high-throughput and isolation efficiency [5,6]. Although they have been successful, there is always a trade-off between the efficiency and throughput of these platforms. Based on the separation mechanism, microfluidic cell isolation techniques can be categorized into active and passive methods [6].

Active methods require an externally applied force so that cells are separated due to their different optical [7], electrical [8], or magnetic [9] properties. Some examples of active methods are dielectrophoresis (DEP) [10], acoustophoresis [11], and magnetophoresis [12]. Although active methods have been proven to be useful in various biological applications, the low-throughput, complex mechanism, and high fabrication costs have limited their widespread applications in most cases.

Passive methods rely on label-free separation of cells using their physical properties without any externally applied force field. Some passive separation methods include deterministic lateral displacement (DLD) [13], pinch flow fractionation (PFF) [14], hydrodynamic filtration [15], and inertial migration [16].

Among passive cell separation techniques, inertial microfluidics has attracted much attention in recent years. The efficiency of inertial microfluidic for cell separation depends on inertial migration and hydrodynamic forces. Inertial migration is a function of the geometrical parameters of the device, while hydrodynamic forces separate particles based on their physical properties, such as density [17], size [18], or deformability [19].

Segre and Silberberg [20,21] were the first to report the inertial migration effect by showing that randomly incoming dispersed particles in a circular tube with a radius of R, can laterally migrate toward the channel walls and form a ring-shaped annulus with a radius of 0.6 R at the outlet. This observation led many researchers to investigate the physics of this phenomenon [22,23]. Particle lateral migration is affected by the shear gradient lift force, which moves the particles toward the tube walls. The wall induces a lift force that pushes the particles through the tube centerline and prevents them from getting close to the walls. As a result, particles reach a certain equilibrium position in the tube’s cross-section depending on the magnitude of these forces, flow velocity, and particle’s physical properties [24]. Due to difficulties with particle separation at the outlet of a circular tube, several studies have investigated particle inertial migration in non-circular channels [25,26,27]. In a square microchannel, eight stable particle equilibrium positions (corners and midline of channel edge) exist in relatively low Reynolds number flows (*Re* < 100) [25]. However, increasing the Reynolds number (*Re* > 500) will reduce the equilibrium positions to four, in which particles are focused at microchannel corners [25]. In a straight rectangular microchannel, randomly dispersed particles with *a_p_/D_h_* ~ 0.1 (where *a_p_* is particle diameter and *D_h_* is hydraulic diameter of the channel) align in four equilibrium positions where shear gradient lift force and wall induced lift force balance each other [27]. According to recent studies, in a spiral microchannel, particles are focused in one single equilibrium position close to the inner wall of the microchannel while smaller sized particles continue to recirculate by the effect of the secondary Dean flow [16,28].

Recently, many studies have been performed to optimize separation by increasing throughput, separation efficiency, and resolution in spiral microchannels [29,30]. Some studies have developed numerical calculations for a better understanding of the inertial focusing mechanism [31,32]. Some studies have been investigated to modify the microchip geometry to optimize the flow rate and isolation efficiency. Label-free tumor cell separation from whole blood has been conducted using a double spiral microchannel device with an 88.5% tumor recovery rate as well as 92.28% recovery for blood cells [33]. Higher particle isolation efficiency was reached by introducing a spiral microchannel with trapezoidal cross-section [34,35] and ordered micro-obstacles [36]. However, the complex fabrication of trapezoidal cross-section and micro-obstacles has limited the wide application of this kind of geometry in microfluidic platforms. Our group proposed a spiral microchannel with a stair-like cross-section for size-based particle separation. Using equilibrated vortices present in the spiral microchannel, it was observed that there is a size-dependent threshold for flow rate for separating each specific particle [37].

This work overcomes the limitations of previous methods by presenting a passive microfluidic approach for high-throughput continuous isolation of WBCs from whole blood in a spiral microchannel with U-shape cross-section using size-dependent inertial migration. The proposed method uses secondary Dean drag force to move smaller cells away and inertial forces to equilibrate larger cells through the spiral microchannel. One of the advantages of the U-shaped cross-section is that target cells can be collected from separate outlets in discrete cell streams without any restriction on the channel length. The U-shaped cross-section also avoids the re-circulation of smaller particles through the entire cross-section. Hence, it removes certain drops in isolation efficiency due to the mixing problems of conventional rectangular cross-sections. Unlike previous microfluidic devices with rather complex geometries and cross-sections, e.g., microchannels with trapezoidal cross-section, or spiral microchannels with ordered micro-bars, our proposed spiral microchannels with the U-shaped cross-section are easy to fabricate. Also, we have developed a novel numerical algorithm to calculate the inertial forces exerting on particles to optimize the proposed device’s cell separation capability that can be used for similar inertial focusing methods for future works. It also further eliminates the time-consuming process of trial and error to find the optimized design of the device. Based on numerical results, we select an optimized geometry regarding the proper placement of the secondary vortices and their primary role in carrying the target cells to proper microchip outlets. Utilizing this method, high separation efficiency for WBCs and high removing ratios for RBCs and platelets were achieved using the fabricated parallel spiral microchannels with a U-shaped cross-section. It was reached under a high-throughput (as high as 6 mL/min), high-capacity (360 mL sample in 1 h) label-free cell sorting without the need for any significant pre-processing or post-processing. Single-layer spiral microchannel with the U-shaped cross-section, which is a modified version of a spiral microchannel with rectangular cross-section, has the following advantages: (i) Strong commercialization potentials due to simplified fabrication methods; (ii) Low fabrication costs and efforts; (iii) A wide range for the separation of cells/particles with different sizes. Such an optimized microfluidic device has great potential for biological cell separation in clinical applications.

## 2. Design Principle

In straight channels, shear-induced lift force resulting from the parabolic nature of the velocity profile in a Poiseuille flow tends to move the dispersed particles away from the center of the microchannel toward the channel walls. As the particle gets closer to the walls, an asymmetric wake around the particle forms a wall-induced lift force that prevents the particles from getting close to the walls and directs them away from the walls toward the channel center [38]. Hence, the particles are focused in equilibrium positions in narrow bands where these two opposite forces are equal [20,21,25,28,39,40]. The overall magnitude of the inertial forces which cause the lateral migration of the particles in a straight microchannel can be estimated by Equation (1) [41]:

(1) $$F_{L}=\rho G^{2}C_{L}\left(Re,\;x_{p}\right)\;a_{p}{}^{4}$$

where ρ is the density of the fluid, G is the fluid shear rate ($G=2U_{f}/D_{h})$, $U_{f}$ is the average flow velocity, $C_{L}$ is a non-dimensional lift coefficient which is a function of Reynolds number and particle position in the channel’s cross-section, U is the maximum velocity in the microchannel, $D_{h}$ is the hydraulic diameter of the microchannel and *a_p_* is the particle diameter. The magnitude of $C_{L}$, thus, $F_{L}$ starts from zero in the channel centerline reaches a maximum value, and then goes back to zero again around 0.2$D_{h}$ away from the channel wall, which is considered as the equilibrium position of particles [41]. Beyond this distance, $C_{L}$ becomes negative in sign showing the dominant effect of wall-induced lift force and further increases in magnitude by moving toward the walls [27].

Fluid flow in curvilinear channels experiences radially outward centrifugal acceleration resulting in the shift of maximum velocity toward the outer wall of the channel, leading to the formation of two counter-rotating vortices known as Dean vortices in the top and bottom halves of the channel cross-section plane [42,43]. The strength of these two identical vortices can be described using the dimensionless Dean number (*De*):

(2) $$De=\frac{\rho U_{f}D_{h}}{\mu }\sqrt{\frac{D_{h}}{2R}}=Re\sqrt{\frac{D_{h}}{2R}}$$

where ρ is the fluid density, $U_{f}$ is the average flow velocity, μ is the fluid viscosity, $D_{h}$ is the hydraulic diameter, R is the flow path curvature, and Re is the flow Reynolds number. According to Stokes law, the applied drag force on a single smooth spherical particle (larger than 1 µm in diameter) in a laminar flow can be calculated using Equation (3) [44]:

(3) $$F_{D}=6\pi \mu \;r_{p}V$$

where μ is the fluid viscosity, $r_{p}$ is the particle radius, and *V* is the particle velocity.

Assuming Stokes law, particles are affected by the drag force due to the presence of the secondary flow in the microchannel, which can be described as:

(4) $$F_{D}=3\pi \mu \;U_{Dean\;}a_{p}$$

where $a_{p}$ is the particle diameter. Ookawara et al. [45] numerically investigated Dean flows in a rectangular curved microchannel and presented a correlation, in the form of a power function, for estimating the average dean flow velocity. They conducted their simulations based on using the SIMPLE algorithm with a second-order upwind scheme in the Fluent software with boundary conditions of uniform velocity inlet and pressure outlet for the microchannel inlet and outlet, respectively. Based on their formulation, the average Dean velocity can be expressed by Equation (4):

(5) $$U_{Dean}=1.8\times 10^{-4}\;De^{1.63}$$

Thus, the drag force caused by the secondary Dean vortices exerting on the particles can be estimated by:

(6) $$F_{D}=5.4\times 10^{-4}\;\pi \mu \;De^{1.63}\;a_{p}$$

The inertial forces and the Dean drag force act in the same direction on the particles near the outer wall while particles near the inner wall experience these two forces in opposite directions. According to Equations (1) and (5), the ratio of the inertial lift force to the Dean drag force (*F_L_*/*F_D_*) is proportional to $\;a_{p}{}^{3}$, therefore, as particles become larger, the inertial lift force exerting on them becomes greater than the Dean drag force and vice versa for small particles. Hence, in a spiral microchannel, larger particles tend to stay in equilibrium positions near the microchannel inner wall, while smaller ones are constantly circulated by the secondary flow.

In this paper, we take advantage of the simultaneous acting of inertial forces and Dean drag force on the blood cells in a spiral microchannel to develop a high-throughput device that can isolate WBCs from whole blood without the needs of blood lysis, cell labeling, centrifugation, and other chemical or physical pre-processing techniques on the sample.

## 3. Materials and Methods

### 3.1. Device Fabrication

**Figure 1:**
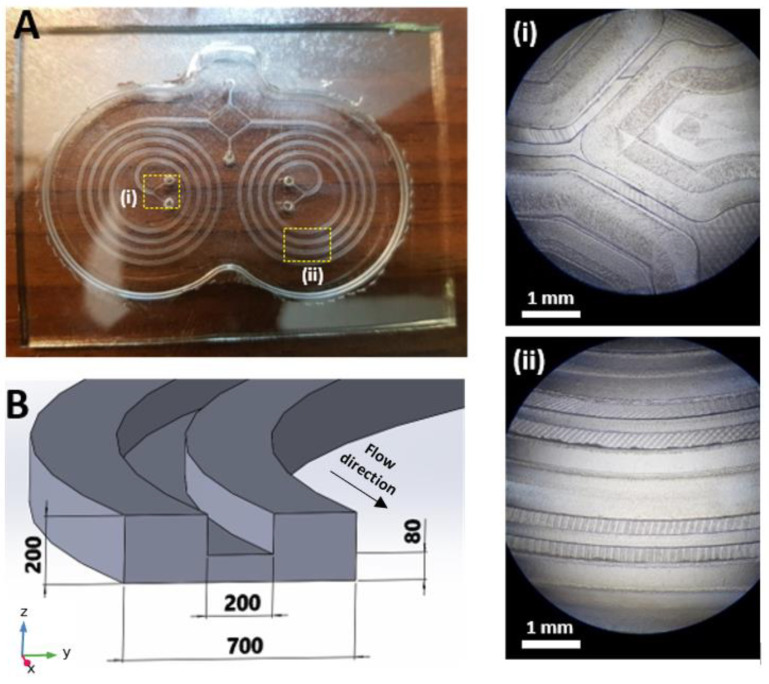
(**A**) The 4-loop parallel spiral microchannels fabricated in PDMS with the corresponding mold preview. (**i**) The magnified view of section (**i**). (**ii**) The magnified view of section (**ii**). (**B**) Schematic of the cross-section of the spiral microchannel (The dimensions are in µm).

Micromilling is a sub-branch of micromachining which has been widely used to fabricate a wide range of microfluidic devices. The advantages of using this technique are faster fabrication, straightforward process, easier user interface, and the ability to manufacture complex geometries, which makes this method an ideal choice in rapid prototyping microfluidic platforms for testing, validation, and research. Unlike other means of fabrication such as PDMS casting, etching, etc., micromilling can be employed on various types of materials and can support the fabrication of multi-level and complex structures. Moreover, micromilling devices, such as CNC machines can be found nearly in any university or workshop and are commonly used in manufacturing processes. PMMA (polymethyl methacrylate) material is mainly used in microfluidics due to its excellent characteristics, such as biocompatibility, good strength, low cost, and optical properties. In this study, the master mold was manufactured by micromilling on a PMMA substrate by Dahlih MCV-1020A milling machine which has an accuracy of 2 µm in the 3 moving directions. Since the microchannel geometry consists of two sets of levels with different heights, several layer cuts are needed, including a couple of rough cuts, and two final cuts with the depths of 1 mm, 120 µm, and 200 µm, respectively. In order to cut the microchannels on the PMMA substrate, 3 different micromilling bits with different sizes were used. A 6 mm diameter 4-flute endmill cutting with a spindle speed of 3000 rpm and cutting feed rate of 100 mm/min was used for starting rough cuts, a 0.8 mm diameter 2-flute endmill cutting with a spindle speed of 6000 rpm and cutting feed rate of 100 mm/min was used for cutting the spaces between the spiral loops, and a 0.1 mm diameter 2-flute endmill cutting with a spindle speed of 7000 rpm and cutting feed rate of 50 mm/min was used for carving the space above the cross-section passway. Once the PMMA mold was ready, we proceeded to the next step. The microchip was fabricated using the standard soft lithography method. Briefly, degassed PDMS (polydimethylsiloxane), previously mixed in a 10:1 pre-polymer base to the curing agent, was cast onto the master mold and baked in a vacuum oven for 4 h at 75 °C. Cured PDMS layer with embedded channels was peeled off, and inlets and outlets holes were punched using a 2 mm biopsy punch. Finally, the PDMS layer was irreversibly bonded to a thick standard glass slab with an oxygen plasma and subsequently baked for 2 h at 85 °C to further improve the bonding. Silicone tubes with an outer diameter of 2 mm were press-fitted into the channel inlets and outlets. The device consists of two parallel 4-loop spiral geometry with 4 inlets, and 2 shared inner and outer outlets. The cross-section of the channels is a U-shape geometry with 700 µm width and 200 µm height, and the two sections of the channel are connected by a 200 µm × 80 µm passway as shown in Figure 1B.

Each spiral microchannel has an initial radius of curvature of 6 mm and a distance of 1.9 mm between two successive loops. The total length of each spiral is approximately 17.6 cm (Figure 1A).

### 3.2. Numerical Simulation

The main idea of simulation is to calculate the forces exerting on the cells in the fluid domain. Since the cells’ size is relatively comparable to microchannel dimensions (14 µm vs. 200 µm), the effect of cells on the fluid domain cannot be ignored. In terms of investigations on the particle lateral migration, most theoretical examinations involve using simplified models, such as circular tubes, parallel plates, and negligible particle diameters (a/H << 1). These models manage to approximate the fluid domain by treating the particle as a dimensionless point with a point-force applying to it, hence failing to take the effect of the particle on the fluid domain into the account. Originally, Di Carlo et al. [31] developed an algorithm to overcome these limitations and calculate the inertial forces exerting on particles with diameters comparable to that of the microchannel dimensions in a straight rectangular microchannel. The main drawback was that it was limited to conventional rectangular cross-section microchannels. However, in this study, we further developed the algorithm so that we could assess the performance of arbitrary complex microchannel cross-sections, hence extending it to investigate the viability of our U-shaped cross-section microchannel on isolating particles with different sizes. Several other useful information can be collected while using this algorithm as a simulation approach, such as the threshold flow rate estimation in which smaller particles begin to migrate toward the outer microchannel section, equilibrium positions of different-sized particles throughout microchannels with complex cross-sections, the amount of shear stress on the surface of particles to evaluate the possibility of cell damage, etc. Herein, we represent our modified version of this algorithm which is developed for a U-shaped cross-section microchannel in the present work as well as supporting more complex cross-sections alongside treating dispersed particles as objects affecting the flow field domain. Also, in terms of simulations, although blood cells don’t exactly match that of a circular shape, they were treated as smooth solid spherical particles for the sake of simplicity.

Inertial lift force is a function of particle location within the cross-section of a microchannel and flow shear rate around the particle. We managed to calculate this force within the cross-section and investigate the equilibrium positions of particles through the cross-section plane. We took the x-direction as the main flow direction in any arbitrary cross-section throughout the spiral microchannel. The simulation consists of three steps:

(a) A 3D steady laminar flow is solved through the whole spiral microchannel, and the velocity profiles are extracted using COMSOL Multiphysics^®^ software.

(b) A straight channel with a proposed cross-section with a length of 20 times the particle diameter is created.

The velocity profile solved at Step (a) is set as the inlet and outlet boundary conditions, and the flow field is calculated through the entire channel. Linear and rotational velocities of flow on some cross-section points will be used in the next step.

(c) Particle modeled as a solid sphere is put in the middle of the microchannel alongside the x-direction, and its location is set to change in the *yz*-plane in each solution step. Steady-state linear and rotational velocities of the particle are unknown and should be calculated using an iterative trial-and-error process.

**Figure 2:**
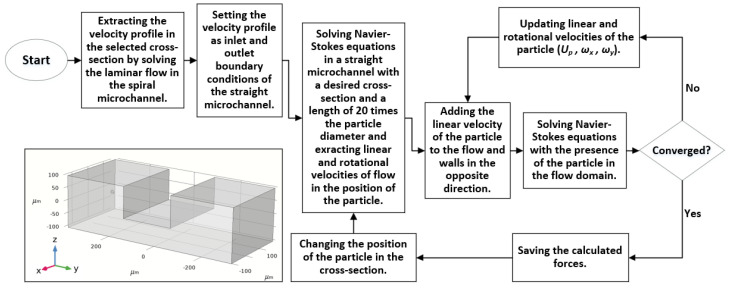
Flowchart of the numerical algorithm used in this study for the calculation of the forces acting on particles. The original algorithm was proposed to investigate particle inertial migration in a straight microchannel [31]. It was later modified to be used in a rectangular spiral microchannel [32]. We extended the original algorithm to run support the corresponding calculations of any other desired spiral microchannel cross-sections.

The flowchart of the proposed algorithm is shown in Figure 2.

The governing equations for fluid flow within the microchannel are continuity and Navier-Stokes equations as follows [46,47]:

(7) ρ∇·u=0

(8) $$\rho \left(u\cdot \nabla \right)u=\nabla \cdot \left[-pI+\mu \left(\nabla u+{\left(\nabla u\right)}^{T}\right)\right]$$

where ρ is the fluid density, ∇ is the differential (Del) operator, u is the flow velocity vector, I is the identity tensor and p is the pressure. The Navier-Stokes equations are solved using the finite element method. We used piecewise-polynomial approximations of equal order to spatially approximate both velocity and pressure [48]. Specifically, piece-wise quadratic (P2 + P2) scheme is chosen to discretize the equations. P2 + P2 scheme uses second-order elements for the velocity field and second-order elements for pressure [48].

To start the algorithm (Step c), we set the initial linear and rotational velocity of the particle equal to the fluid velocities at the exact location of the particle, which was solved in step (b). In fact, these initially estimated velocities are from the that no particle is present in the flow field.

Moving wall boundary condition is applied to channel walls with velocities equal to particle’s linear velocity ($U_{p}$) in the opposite direction (–x):

(9) $$U_{wall}=-U_{p}$$

Channel inlet and outlet are set to the same velocity profile given in step (b) and are updated in every iteration using the particle’s axial velocity as follows:

(10) $$U_{in}=U_{fluid,\;old}-U_{p}$$

Particle is treated as a solid sphere with rotating walls:

(11) $$\vec{U}=\vec{\Omega_{p}}\times \left(\vec{r\;}-\vec{r_{p}}\right)$$

where $\left(\vec{r\;}-\vec{r_{p}}\right)$ represents the location of a point on the particle surface relative to its center with a flow velocity vector of $\vec{U}$, and $\vec{\Omega_{p}}$ represents the particle rotational velocity vector.

Particle rotational and axial linear velocities are updated during the calculations until the axial force, and cross-sectional components of momentum exerting on the particle become less than 1 × 10^−14^ N and 1 × 10^−20^ N·m, respectively, hence, to be considered as a steady-state condition for the particle. Once convergence occurs, calculations will start over with the particle being placed at another location in the *yz*-plane. The following formulas are used to update the velocities:

(12) $$U_{p}{}^{\prime \prime }=U_{p}{}^{\prime }+a_{z}\times \Delta t$$

(13) $$\Omega_{y}{}^{\prime \prime }=\Omega_{y}{}^{\prime }+\alpha_{y}\times \Delta t$$

(14) $$\Omega_{z}{}^{\prime \prime }=\Omega_{z}{}^{\prime }+\alpha_{z}\times \Delta t$$

where superscripts indices (″) and (′) refer to old and new calculated parameters, respectively, $U_{p}$ is the axial linear velocity of the particle, and $a_{x}$, $\alpha_{y}$ and $\alpha_{z}$ are the linear and cross-sectional rotational accelerations of the particle, respectively, which can be found using Newton’s second law of motion.

After the inertial forces are calculated for a specific particle in the whole cross-section, we can investigate whether the particle can be forced to move through the middle passway by the secondary flow field or not, hence characterizing the ability of the microchannel to separate the target cells.

### 3.3. Mesh Independency

**Figure 3:**
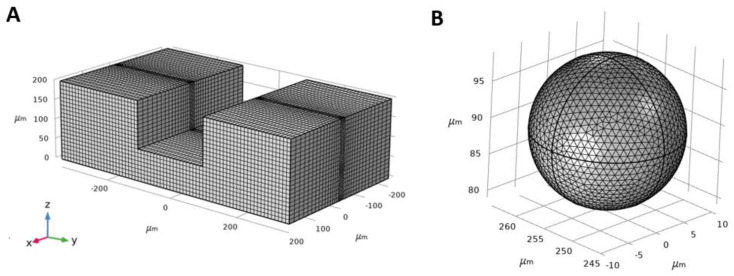
Illustration of the mesh in the fluid domain. (**A**) The domain was separated into two zones in which tetrahedral mesh was used between the particle surface and the microchannel walls with a maximum size of 0.1 × D_p,_ and the structured mesh was used outside the block, including the particle. (**B**) Triangular mesh was used on the surface of the particle with a maximum size of 0.05 × D_p_.

A mixed structured-unstructured type of mesh was used with the presence of the particle in the microchannel (Figure 3A). The microchannel was split into two distinct parts: (i) a block containing the particle with a cross-section similar to the cross-section of the microchannel in which triangular and tetrahedral grids were used for the surface of the particle and the rest of the zone, respectively, (ii) other zones excluding the particle in which structured grids were used. The inertial lift force exerting on the particle in the y-direction throughout the microchannel cross-section was selected as the mesh independence criteria and various mesh resolutions were considered to evaluate grid independence. The results show that the maximum value for different parameters, including the width of the block containing the particle, size of the tetrahedral grids, and size of the triangular grids on the surface of the particle are 5 × D_p_, 0.1 × D_p_, and 0.05 × D_p_, respectively (Figure 3B), in order to preserve less than 0.1% change in the y-component of the lift force with *D_p_* being the particle diameter.

### 3.4. Sample Preparation

Human blood samples were collected using a blood collection tube. 10 mL of blood was mixed with 35 µL ethylenediaminetetraacetic acid (EDTA) solution (7 µL per 2 mL of blood) to prevent blood clotting. This study was approved by the Research Ethics Committees of the Iran University of Medical Sciences (ID: IR.IUMS.REC.1400.285), and informed consent was obtained from the subject involved in the study. In addition, all clinical experiments were conducted in accordance with the ethical principles and related guidelines. Due to the non-Newtonian behavior of blood and high concentration of RBCs in the blood, we diluted the starting sample with DI water. The donor’s blood was measured to be at 45% hematocrit upon donation. Three tubes of the sample were prepared by mixing the whole blood with DI water with the ratios of 1:45, 1:22.5, and 1:9, therefore, the yielding blood samples were measured to be at 1%, 2%, and 5% hematocrits, respectively.

Although in this case, minor RBC lysis may occur due to hemolysis which is caused by osmotic effects of blood dilution, since the main aim of this research is to prepare WBC samples, this effect is ignored. However, in cases of RBC isolation and RBC sample preparation, the usage of saline solutions (i.e., PBS) is recommended in order to boost and preserve RBC recovery.

### 3.5. Experimental Approach

Throughout the tests, the prepared blood samples were pumped through the spirals’ inner inlets as well as an equal flow of DI water as the sheath flow through the outer inlets using two 10 mL syringes with a syringe pump with flows varying from 2 mL/min up to 8 mL/min. Processed samples were collected from inner and outer outlets in silicone tubes, and cell counting was done using a Neubauer chamber.

A total number of 4 distinct tests were done for each flow rate and sample hematocrit to reduce uncertainty and errors caused by devices and cell enumeration. To get well-balanced results, DI water was set to be pumped into the spiral microchannel to wash it out from the remaining blood cells from previous use before running further tests.

Since this research aims to isolate WBCs from whole blood and remove RBCs and platelets from the target outlet (inner outlet), we introduce the terms ‘isolation efficiency’ and ‘removing ratio’ for WBCs and RBCs + platelets, respectively, to evaluate the device performance.

The ratio of the number of WBCs in the inner outlet to the whole number of WBCs in both outlets is reported as the ‘WBC isolation efficiency’ of the spiral microchannel and the ratio of the number of WBCs to the whole number of blood cells in the inner outlet is reported as ‘WBC purity’:

(15) $$\%\mathrm{WBC}=\frac{\#\;\mathrm{of}\;\mathrm{WBC}\;\mathrm{in}\;\mathrm{the}\;\mathrm{inner}\;\mathrm{outlet}}{\#\;\mathrm{of}\;\mathrm{WBC}\;\mathrm{in}\;\left(\mathrm{inner}+\mathrm{outer}\right)\;\mathrm{outlets}}$$

(16) $$\%\mathrm{Purity}=\frac{\#\;\mathrm{of}\;\mathrm{WBC}\;\mathrm{in}\;\mathrm{the}\;\mathrm{inner}\;\mathrm{outlet}}{\#\;\mathrm{of}\;\mathrm{total}\;\mathrm{cells}\;\mathrm{in}\;\mathrm{the}\;\mathrm{inner}\;\mathrm{outlet}}$$

Moreover, the removing ratio of RBCs and platelets are obtained using the equations below:

(17) $$\%\mathrm{RBC}=\frac{\#\;\mathrm{of}\;\mathrm{RBC}\;\mathrm{in}\;\mathrm{the}\;\mathrm{outer}\;\mathrm{outlet}}{\#\;\mathrm{of}\;\mathrm{RBC}\;\mathrm{in}\;\left(\mathrm{inner}+\mathrm{outer}\right)\;\mathrm{outlets}}$$

(18) $$\%\mathrm{Platelets}=\frac{\#\;\mathrm{of}\;\mathrm{Platelets}\;\mathrm{in}\;\mathrm{the}\;\mathrm{outer}\;\mathrm{outlet}}{\#\;\mathrm{of}\;\mathrm{Platelet}\;\mathrm{in}\;\left(\mathrm{inner}+\mathrm{outer}\right)\;\mathrm{outlets}}$$

## 4. Results and Discussion

### 4.1. Device Design for Cell Separation

**Figure 4:**
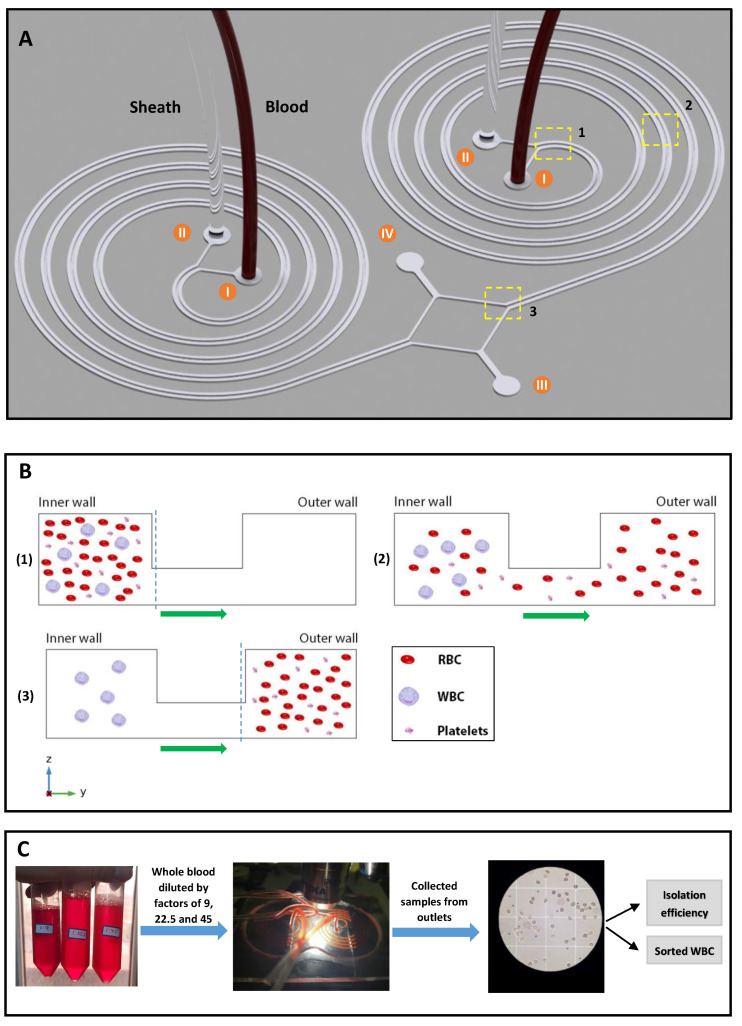
(**A**) 3D schematic of the proposed spiral microchip in this study. The design includes two parallel U-shaped cross-section spiral microchannels that consist of: (I) two inner inlets for the injection of blood samples, (II) two outer inlets for the injection of sheath fluid, (III) shared outer outlet for the extraction of RBCs and platelets, and (IV) shared inner outlet for the extraction of WBCs. (**B**) Larger blood cells, i.e., WBCs, affected by dominant inertial forces are focused along the inner microchannel wall. In comparison, smaller blood cells, i.e., RBCs and platelets, are forced to migrate toward the outer microchannel section by the effect of the secondary flow (flow direction is shown in green arrows), which in turn stops the smaller cells from getting back to the inner microchannel section. (**C**) The overall process of WBC sorting and enumeration. Blood cell count is done using a Neubauer chamber, and the resulting WBC isolation efficiency is calculated using the fractional ratio of blood cells in specific outlets to the total count of blood cells in both outlets.

The concept of cell separation in this method is illustrated in Figure 4. The U-shaped cross-section design proposed in this study can keep large particles, such as WBCs, in the inner microchannel section, while forcing smaller cells, like RBCs and platelets, to migrate to the outer microchannel section through the middle passway without recirculating and coming back to the inner section of the microchannel. Additionally, the proposed U-shaped cross-section eliminates the re-circulation and mixing problems of conventional rectangular cross-sections. In order to reach a decent isolation flow rate, two identical spiral microchannels are set as parallel to each other, with inner outlets and outer outlets being merged into a single inner and outer outlet, respectively. At the end of the experiment, WBCs ($a_{p}\;\sim \;14\;\mu m$) are collected from the inner outlets and RBCs ($a_{p}\;\sim \;7\;\mu m$) and platelets ($a_{p}\;\sim \;3\;\mu m$) are collected from outer outlets. Due to the size and flow dependence of the forces exerting on the blood cells, we managed to investigate the effect of flow rate and cell concentration on the WBC isolation efficiency and RBC and platelets removing ratios in the spiral microchannel to find the optimum flow rate.

### 4.2. Validation of the Numerical Simulations

**Figure 5:**
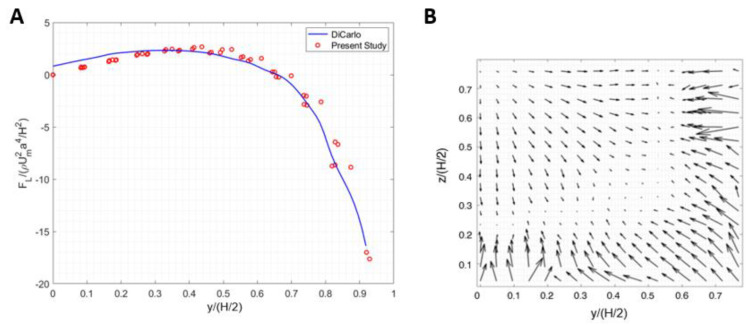
(**A**) Calculated inertial force in a straight channel at z = 0 using the algorithm in the present study versus inertial force reported by Ref. [31]. (**B**) Calculated inertial force field acting on a 10 µm particle in the present study.

The microfluidic device proposed by Di Carlo et al. [31] was a straight channel with a 50 µm × 40 µm rectangular cross-section, and the diameter of the particle was 10 µm in a flow with *Re* = 80. In order to validate our algorithm, inertial lift forces acting on a 10 µm particle were calculated in the same straight microchannel (Figure 5A). Particle equilibrium positions are located in the areas where the force vector approaches zero value, approximately 0.6 times of half channel width. As can be seen in Figure 5B, our simulations are quite consistent with Di Carlo’s results [31] and demonstrate the same equilibrium regions. Next, we investigated the ability of the developed algorithm in this study by performing the calculations on the U-shaped cross-section microchannel.

### 4.3. Simulation of the Secondary Flow in the Spiral Microchannel

**Figure 6:**
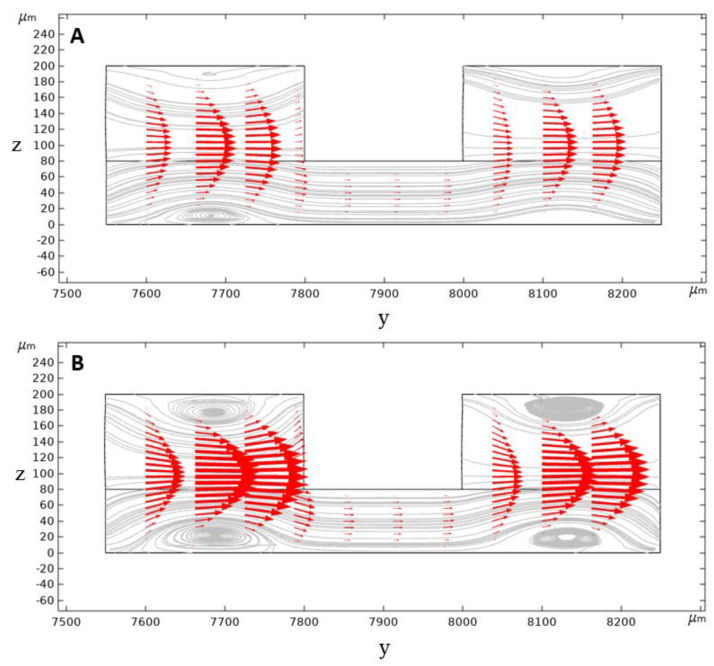
Calculated secondary flow streamlines and velocity vectors in the second loop of the designed spiral microchannel cross-section at x = 0. Velocity vectors are being directed from the inner wall to the outer wall in the middle passway with: (**A**) 2 mL/min, (**B**) 3 mL/min inlet flow rate (Note that in this figure the origin of y-direction lies on the origin of the spiral microchannel).

In a conventional rectangular cross-section spiral microchannel, pressure gradient results in the formation of the secondary flow consisting of two similar counter-rotating Dean vortices. However, in our geometry, the secondary flow consists of four Dean vortices locating at the top and bottom of the inner and outer channel sections. Dean vortices’ strength is increased by further increasing the flow rate and the Dean number and, therefore, the vortex cores start to appear (Figure 6).

Contours of velocity magnitude and the schematic of the flow velocity profile in this study are shown in Figure 6A,B. As can be seen, there are two locations of maximum velocity in the inner and outer section of the microchannel, with the magnitude of maximum velocity in the inner section being higher than the outer one. This difference generates a pressure gradient that causes the secondary flow’s streamline to be directed from the inner section toward the outer microchannel section. Thus, the secondary flow prevents the migrated particles from returning to the inner microchannel section through the middle passway. This option removes the possibilities of cell circulation through the entire cross-section and increases the isolation efficiency.

**Figure 7:**
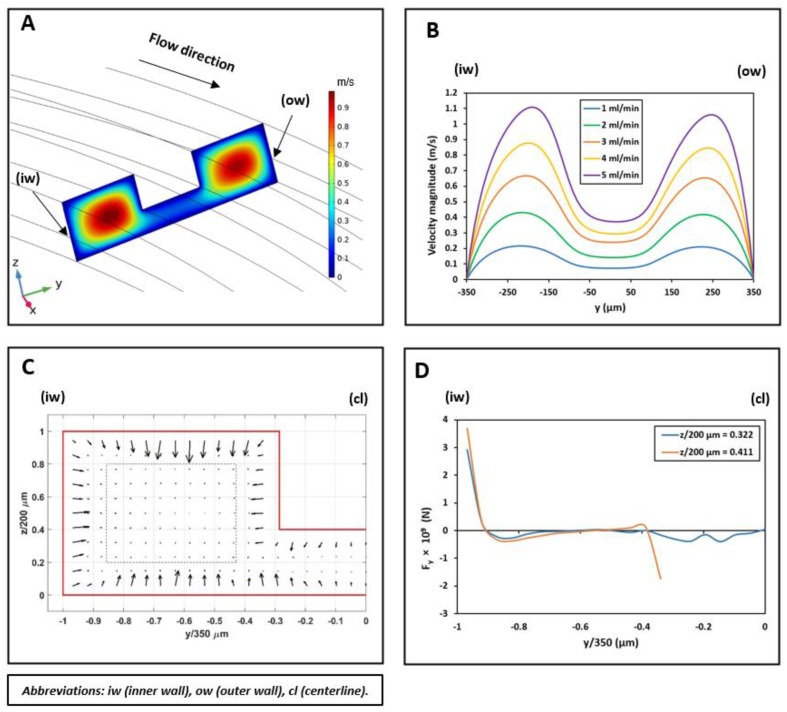
(**A**) Contour of velocity magnitude in the spiral microchannel at the second loop at x = 0 with an inlet flow rate of 3 mL/min. (**B**) Velocity magnitude profile with the change in inlet flow rates at z = 40 µm (z = 0 indicates the bottom wall of the microchannel and y = 0 indicates the centerline of the microchannel cross-section (cl), this difference in the maximum velocity magnitude results in the streamlines being directed from the inner microchannel wall (iw) toward the outer microchannel wall (ow). (**C**) The calculated inertial force field acting on a 10 µm particle in the inner section of the spiral microchannel with an inlet flow rate of 5 mL/min. Equilibrium positions are shown as blue dashed lines in the microchannel cross-section. (**D**) The inertial force applying to a 10 µm particle alongside y-direction at z = 64.4 µm (z/200 µm = 0.322) and z = 82.2 µm (z/200 µm = 0.411). As can be seen, the inertial force at the beginning of the passway is much lower than the wall-induced inertial force. The Dean drag force can overcome this inertial force, therefore, allowing the particles smaller than 10 µm to move toward the outer section of the spiral microchannel.

The calculated inertial force field acting on a 10 µm particle in the U-shape channel is shown in Figure 7C. According to the simulation results, 10 µm particles migrate from the center of the microchannel’s inner section toward the microchannel walls. As the particles get closer to the walls, a rather large inertial force stops them from further approaching the walls. The particles tend to reach equilibrium positions where the total amount of inertial forces exerting on them is zero; these positions are shown as blue dashed lines.

The U-shaped microchannel’s capability to isolate the particles depends on the value of the exerting the inertial forces and the Dean drag force on the particles near the middle passway. Suppose the Dean drag force overcomes the inertial forces. In that case, the particles migrate to the outer microchannel section through the middle passway and, due to the direction of the streamlines, they cannot come back to the inner section. The inertial lift force profile applying to a 10 µm particle alongside the y-direction is given in two different microchannel heights at the beginning of the middle passway in Figure 7D. According to the calculation results for 5 mL/min flowrate, the Dean drag force is strong enough to overcome the inertial forces for particles smaller than 10 µm, hence moving them to the outer microchannel section. However, for particles larger than 10 µm, the inertial lift force near the passway walls is strong enough to overcome the Dean drag force to keep these particles in the inner section. We did our calculations based on a 10 µm particle to ensure a safe particle diameter margin in which WBCs ~ 14 µm are isolated from smaller particles (RBCs ~ 7 µm and platelets ~ 3 µm) in the designed spiral microchannel. The optimum flow rate for the isolation of blood cells will be investigated experimentally, which will be discussed in the next sections.

**Figure 8:**
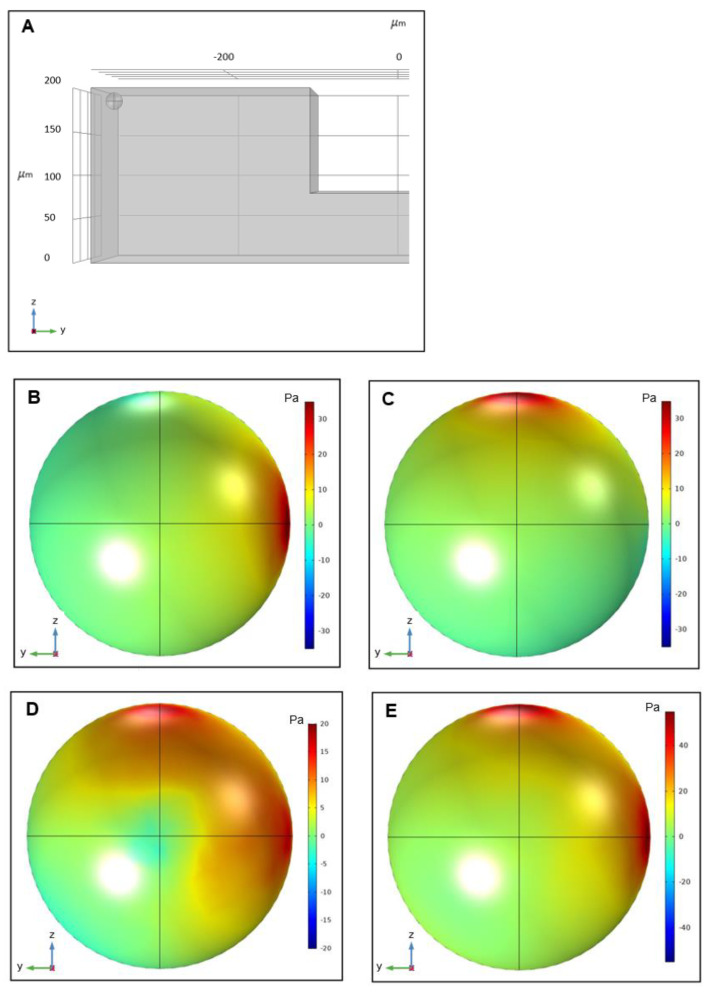
Schematic of (**A**) the critical location for a 14 µm particle in terms of experiencing the maximum shear stress throughout the spiral microchannel, (**B**) the total shear stress exerting on the particle in the x-direction, (**C**) the total shear stress exerting on the particle in the y-direction, (**D**) the total shear stress exerting on the particle in the z-direction, (**E**) the total magnitude of shear stress exerting on the particle. The resulting maximum stress in the x, y, and z-direction was found to be 35 Pa, 35 Pa, and 20 Pa, respectively. Moreover, the maximum value for the magnitude of total shear stress is 45 Pa.

Contours of the total shear stress exerting on a 14 µm particle in the 3 directions can be seen in Figure 8. According to the simulations, the particle is prone to maximum stress in the flow rate of 8 mL/min at the top corner of the cross-section near the microchannel inner wall shortly after the microchip inlet. In the worst-case scenario, the magnitude of the total stress applying to a 14 µm is calculated to be less than 45 Pa. Cell loss and damage due to fluid shear stress is a function of both stress magnitude and exposure time. The less exposure time to a particular shear stress, the less the chances of having cells damaged [49]. The critical magnitude of shear stress in this study for a 14 µm particle which is set to represent a WBC, remains relatively below the known threshold of WBC damaging shear stress [49]. Additionally, the low exposure time of WBCs to these amounts of shear stress, which equals to the amount of time that a single cell manages to pass through the microchannel (about 0.306 s) further lessons the possibility of losing WBCs in the process.

### 4.4. Presence of the Secondary Flow in the Spiral Microchip

**Figure 9:**
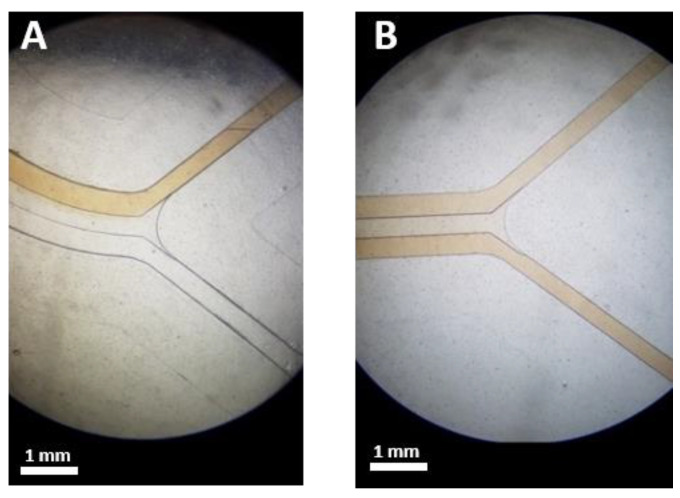
Images of the left side spiral at (**A**) Inlets, (**B**) outlets showing the effect of the secondary flow on mixing the colored fluid throughout the entire microchannel. The colored fluid and DI water were pumped through the microchannel from the inner and outer inlets, respectively.

In order to confirm the presence of the secondary flow in the spiral microchannel, two syringes of fluid were prepared. One syringe was filled with a colored fluid, while the other one was filled with DI water. The colored fluid was introduced into the spiral microchannels through the inner inlets, and DI water was set to enter through the outer inlets. The two syringes with 10 mL capacity were driven at 5 mL/min (2.5 mL/min per spiral) (*Re* = 81.7, *De* = 11.2) using a syringe pump, and images of the flow streams were captured at the inlets and outlets of the left side spiral. As shown in Figure 9, the colored fluid filled the entire section of the microchannel at the outlet bifurcation, showing the Dean vortices’ effect to mix the flow in both the inner and outer sections of the spiral microchannel.

### 4.5. The Effect of the Flow Rate

To investigate the effect of the flow rate on the separation of blood cells, blood sample with the hematocrit of 1% was injected into the microchip through the inner inlets with 2–8 mL/min flowrate (with 1 mL/min step), and DI water was injected through the outer inlets as the sheath flow with the same flow rate.

The corresponding Reynolds and Dean number for various testing flow rates for the microchip are listed in Table 1.

**Figure 10:**
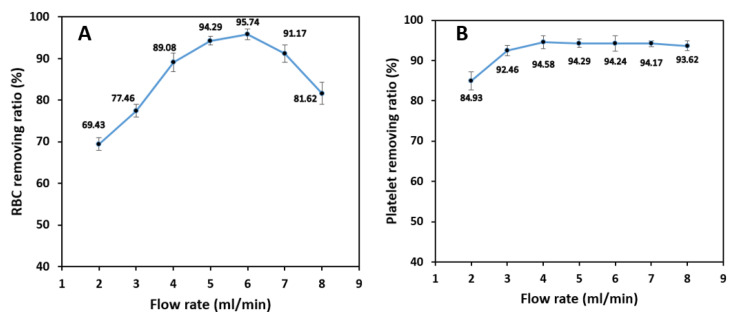
The effect of the sample inlet flow rate on the removing ratio of (**A**) RBCs, (**B**) platelets for 1% blood hematocrit sample. RBC removing ratio is the most sensitive to changes in inlet flow rate with a maximum value at 6 mL/min. The removing ratio of platelets is the least dependent on the flow rate and they can be extracted from the shared outer outlets with high removing ratios.

As shown in Figure 10A, RBCs’ removing ratio reaches a maximum value of 95.7% by increasing the blood sample flow rate up to 6 mL/min and then decreases with further flowrate increment.

The initial expectation would be that for sample flow rates higher than 6 mL/min, small cells do not have enough time (length) to migrate from the inner to outer section due to higher linear velocity. However, further investigations show that increasing the flow rate increases the inertial force more than the Dean drag force. The resultant force stops the majority of the RBCs from migrating through the middle passway, and, hence, the overall efficiency decreases. Additionally, local perturbations in the place of the outlet bifurcation negatively affect the isolation efficiency and removing ratios, which causes the cells to exit through the wrong outlets and further reducing the device’s performance.

Unlike RBCs and WBCs in which inertial and Dean drag forces acting on them compete with each other all the time, platelets are always affected by Dean drag force due to their very small diameter. The removing ratio for platelets reaches a maximum value of 94.6% at a flow rate of 4 mL/min and remains almost constant up to 8 mL/min. Since the inertial forces and the Dean drag force are proportional to the 4th and 1st power of the particle diameter, respectively, the value of the inertial forces applying to the platelets are negligible, and they accumulate in the outer section of the spiral microchannel with a high average removing ratio (Figure 10B).

**Figure 11:**
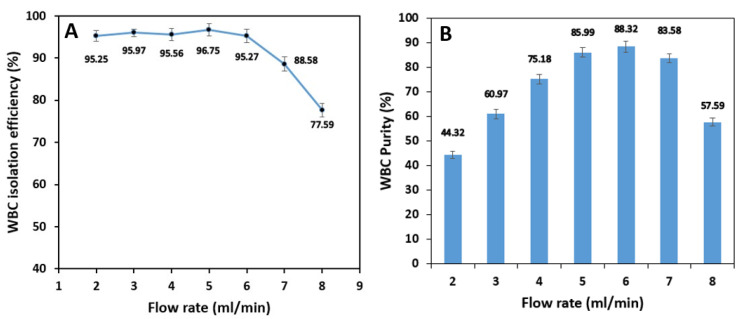
The effect of the sample inlet flow rate on WBC: (**A**) isolation efficiency, and (**B**) purity for a 1% blood hematocrit sample. The WBC isolation efficiency experiences a massive drop in higher flow rates due to the existence of high-strength dean vortices which force the WBCs to migrate to the outer microchannel wall. The outlet samples are the purest with flow rates of 4, 5, and 6 mL/min. The higher the removing ratio of RBCs and platelets, the higher WBC purity.

Test results show that the isolation efficiency of WBCs from whole blood is almost stable up to a flow rate of 6 mL/min with 96.8% maximum efficiency at 5 mL/min. Increasing the sample flow rate has virtually no significant effect on the isolation of WBCs because the inertial forces have the leading role in keeping the cells in the inner section of the spiral microchannel. However, further increase in flow rate from 6 mL/min increases the Dean vortices’ strength, which tends to move the white blood cells to the outer section and decreases the WBC isolation efficiency (Figure 11A). According to Figure 11B, WBC purity increases significantly from 44.3% at 2 mL/min to 88.3% at 6 mL/min by increasing the flow rate since the removing ratio of RBCs has also the same trend with respect to flow rate. A further increase in the flow rate results in a fall in purity since RBCs and platelets manage to remain in the inner section within the strong vortices.

Since RBC removing ratio is highly dependent on the inlet flow rate, the overall criteria of the success of the spiral microchip on isolating the WBCs from whole blood must be selected based on the best isolation efficiency of WBCs and removing ratio of RBCs at the same time. As a result, for a 1% hematocrit blood sample, our spiral microchip can isolate WBCs from whole blood with 96.8% efficiency at the inlet flow rate of 6 mL/min with a WBC sample purity of 88.3%. Moreover, the removing ratios of RBCs and platelets, in this case, are 95.8% and 94.2%, respectively at this flow rate.

Using flow rates of more than 8 mL/min is not applicable due to cell damage possibilities. Additionally, higher flow rates may break up the PDMS-glass slide bonding and are dangerous for test operators, so continuing the tests with flow rates of more than 8 mL/min was not possible.

### 4.6. The Effect of Hematocrit

The same methods and tests were applied to blood samples with 2% and 5% hematocrit to find the effect of the hematocrit on the efficiency of cell separation.

**Figure 12:**
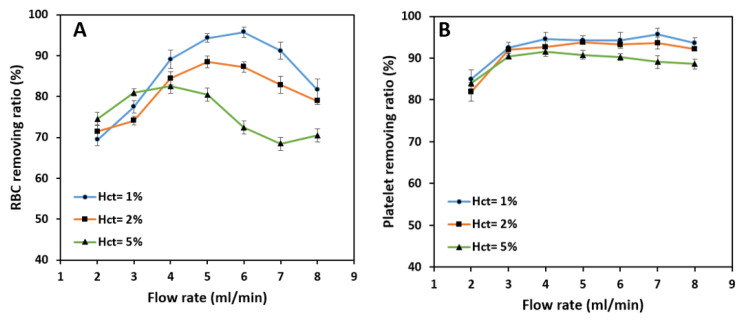
The effect of blood hematocrit on the removing ratio of (**A**) RBC, (**B**) Platelets. The removing ratio mainly decreases with an increase in blood hematocrit due to a rise in the effect of cell-cell interaction. While RBC removing ratio is highly dependent on blood hematocrit, the removing ratio of platelets does not seem to change very much with a change in blood hematocrit.

As shown in Figure 12A, the general trend of the removing ratio of RBCs remains the same for three tested blood hematocrits. RBC removing ratio peaks at 88.4% and 82.5% at 4 mL/min and 5 mL/min flow rates for 2% and 5% hematocrit, respectively, which is considered as a significant drop in the RBC removing ratio compared with 95.7% of 1% hematocrit blood sample at 6 mL/min flow rate. At relatively low flow rates, the difference in removing ratios is negligible for three hematocrits. However, in the 3–7 mL/min flow rate band, the ratio drops with the increase in hematocrit, and this difference tends to grow by increasing the flow rate up to 7 mL/min. The decrease in removing ratio with an increase in hematocrit is due to the higher concentrations of RBCs in higher blood hematocrits that raise the effect of cell-cell interaction force alongside the inertial forces and Dean drag force, which further degrades the overall removing ratio. Platelets typically focus in the outer microchannel section and can be extracted from the outer outlet at relatively high removing ratios Figure 12B. The effect of hematocrit on the isolation is relatively low in this case. It peaks at a value of 93.8% at 5 mL/min and 91.6% at 4 mL/min for 2% and 5% blood hematocrit, respectively, which is pretty much close to 94.2% extraction at 6 mL/min for 1% blood hematocrit. The reason is that the platelets are quite small, and cell-cell interactions are negligible. Hence, the primary force acting on the platelets is the secondary Dean drag force which carries them to the outer section of the spiral microchannel through the middle passway.

**Figure 13:**
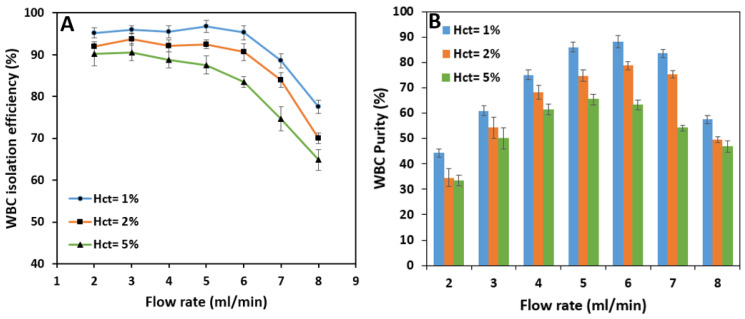
The effect of blood hematocrit on WBCs’: (**A**) isolation efficiency, (**B**) purity. Separation efficiency mainly decreases with an increase in blood hematocrit due to the rise in the effect of cell-cell interaction in higher flow rates. Sample purities for lower hematocrits are mainly higher since to amount of RBCs in the diluted sample is lower. WBC purity is higher at the inlet flow rates of 5, 6, and 7 mL/min due to an increase in RBCs removing ratio.

According to Figure 13A, the overall isolation efficiency of WBCs decreases as hematocrit increases. The efficiency starts at relatively high amounts at the flow rate of 2 mL/min; i.e., 92% and 90.2% for 2% and 5% hematocrits which are lower than the 95.2% efficiency value reached by 1% blood hematocrit.

As the flow rate increases, the reduction in isolation efficiency becomes noticeable for higher hematocrits. In terms of purity, higher sample hematocrits are less pure than the sample with 1% hematocrit due to the fact that more RBCs and platelets are present in these samples. The WBC outlet sample has a maximum purity of 78.8% at 6 mL/min and 65.5% at 5 mL/min flow rate for 2% and 5% hematocrits, respectively (Figure 13B). These figures are pretty much lower than the maximum purity for 1% hematocrit which is 88.3%. Therefore, increasing the sample hematocrit has a negative effect on WBC purity.

The three best overall performances of the microchip tested at different flow rates and hematocrits are listed and summarized in Table 2.

**Figure 14:**
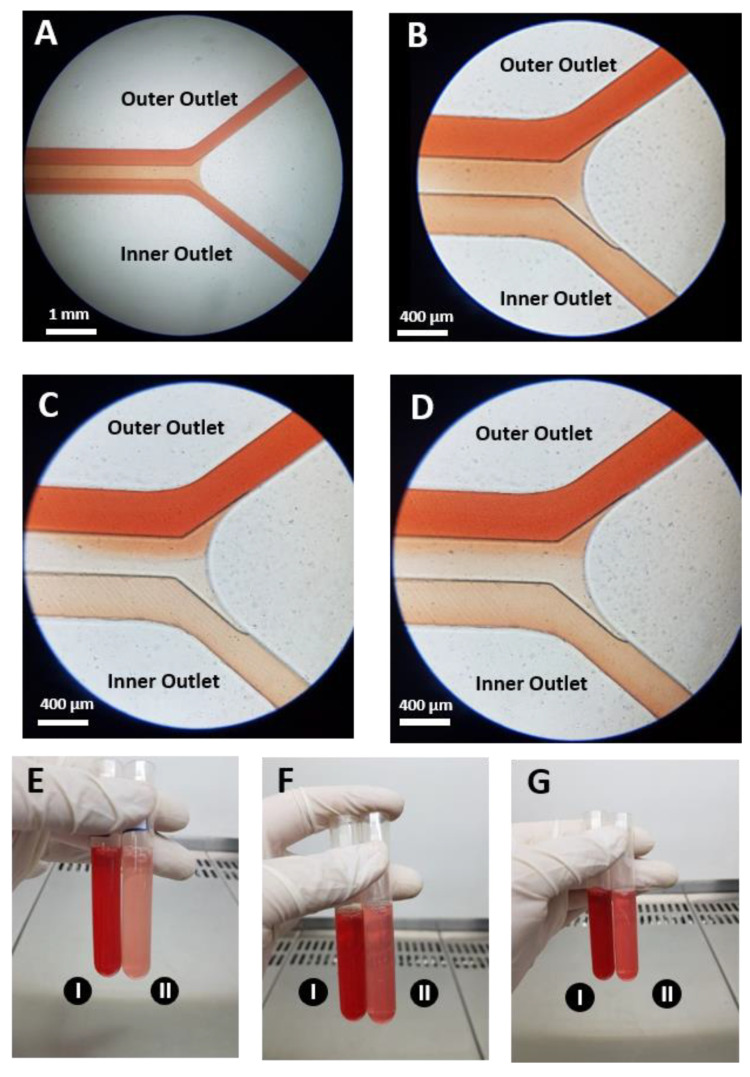
Illustration of the flow in outlet bifurcation in the left spiral for 1% hematocrit blood sample with (**A**) 2 mL/min (**B**) 4 mL/min (**C**) 6 mL/min and (**D**) 7 mL/min flow rates and collected blood samples with (**E**) 1%, (**F**) 2% and (**G**) 5% hematocrit for 6 mL/min flow rate from microchip’s: (I) outer outlets and (II) inner outlets. As can be seen, for constant sample hematocrit, increasing the flow rate reduces the concentration of RBCs in the inner outlets, which results in an increase in WBC WBC and isolation efficiency and RBC removing ratio. For constant flow rates, reducing the sample hematocrit (i.e., higher dilution factors) further reduces the concentration of RBCs in the inner outlets, thus, the collected sample from the inner outlet looks more transparent in comparison to higher sample hematocrits.

With all things considered, optimum results are achieved under a flow rate of 6 mL/min for a 1% hematocrit blood sample. The illustration of flow and collected blood samples from the microchip outlets are shown in Figure 14.

The isolation of WBCs from whole blood has been the main purpose of our spiral microchip. Since the RBC removing ratio is highly sensitive to inlet flow rates, even a slight improvement in removing the red blood cells from the target outlet is a success. Because any sample contains numerous RBCs compared to other blood components (about 1 WBC to every 600 RBC), we managed to select the best performance based on the highest value for RBC removing ratio. Yet, we had to sacrifice a small amount of WBC isolation efficiency.

The numerical simulations showed that a 5 mL/min flow rate could force particles smaller than 10 µm to migrate to the outer microchannel section while holding the particles larger than 10 µm in the inner section. Although the simulation settings are for the case of a single solid particle dispersed in a Newtonian fluid without the interference of other particles, the experimental reported results are reasonably acceptable for 1% hematocrit at 5–6 mL/min flow rate.

## 5. Conclusions

This paper proposed a passive cell separating method using two parallel U-shaped spiral microchannels for size-based, label-free and continuous separation of WBCs from whole blood based on cell inertial migration. The spiral microchannel with U-shaped cross-section increases WBC isolation efficiency by avoiding the re-circulation of smaller cells (RBCs and platelets) through the entire microchip, thus eliminating the major drawback of the conventional rectangular spiral microchannel. Numerical simulations were performed to study the flow behavior, secondary flow distribution, and particle migration mechanism in the proposed design. An algorithm was developed to calculate the hydrodynamic forces exerting on particles present in the U-shaped spiral microchannel to further predict the design’s performance before proceeding to the fabrication of the microchip as well as checking the possibility of WBC damage due to the applying shear stress to them. Subsequently, we successfully isolated WBCs from whole blood in our experiments and investigated the effect of flow rate and hematocrit on the WBC isolation efficiency and RBC and platelet removing ratios. The best performance can be achieved for a 6 mL/min flow rate with 95.3% and 88.3% isolation efficiency and purity for WBCs, respectively, and removing ratios of 95.7%, and 94.2% for RBCs, and platelets, respectively, for a 1% hematocrit blood sample. Also, our device can process an amount of 360 mL of 1% hematocrit blood sample in 1 h under its best condition which is considered to be higher than most of the conventional cell separating microfluidics platforms. Additionally, the proposed spiral microchip allows a high-throughput, high-efficiency, non-invasive, size-based label-free WBC separation from whole blood without the assistance of RBC lysis, density gradient centrifugation, cell biomarkers, and chemical treatments on the sample. Most importantly, it can be integrated into more complex microfluidic platforms.

Moreover, we successfully tested the double parallel spiral microchannels. We reached decent isolation results that are already superior to most of the microfluidic cell sorting platforms in case of fast processing time. There is still room for improvement in microchip throughput, which can be done by parallelizing more spiral microchannels while maintaining high performance.

## Figures and Tables

**Table 1:** The microchip’s specifications in terms of sample flow rate, microchannel Reynolds number, and Dean number.

Sample Flow Rate (mL/min)	Sample Flow Rate Per Spiral (mL/min)	*Re*	*De*
2	1	32.68	4.50
3	1.5	49.02	6.75
4	2	63.36	9.00
5	2.5	81.70	7.47
6	3	98.04	13.50
7	3.5	114.38	15.75
8	4	130.72	18.00

**Table 2:** Summary of the spiral microchip best performance results in the isolation of WBC, RBC, and Platelets based on the inlet sample flow rate and hematocrit.

		Sample Hematocrit		
		1%	2%	5%
	**Flow rate (mL/min)**	6	5	4
**WBC**	**Purity (%)**	88.3	74.8	61.5
	**Isolation efficiency (%)**	95.3	92.5	88.8
**RBC**	**Removing ratio (%)**	95.7	88.4	82.5
**Platelet**	**Removing ratio (%)**	94.2	93.8	91.6

## Data Availability

The datasets used and/or analyzed during the current study are available from the corresponding author on reasonable request.
